# PSGL-1 is a novel tumor microenvironment prognostic biomarker with cervical high-grade squamous lesions and more

**DOI:** 10.3389/fonc.2023.1052201

**Published:** 2023-03-08

**Authors:** Yingying Lin, Shan Huang, Yuanjie Qi, Li Xie, Junying Jiang, Hua Li, Zhiwei Chen

**Affiliations:** ^1^ Department of Healthcare, Fujian Maternity and Child Health Hospital, College of Clinical Medicine for Obstetrics & Gynecology and Pediatrics, Fujian Medical University, Fuzhou, China; ^2^ Department of Traditional Chinese Medicine, Fujian Maternity and Child Health Hospital, College of Clinical Medicine for Obstetrics & Gynecology and Pediatrics, Fujian Medical University, Fuzhou, China; ^3^ Department of Gynecologic, Fujian Maternity and Child Health Hospital, College of Clinical Medicine for Obstetrics & Gynecology and Pediatrics, Fujian Medical University, Fuzhou, China

**Keywords:** PSGL-1, cervical cancer, prognosis, tumor-infiltrating-immune cells, immunotherapy

## Abstract

**Background:**

Macrophages secrete many cytokines and chemokines, which can provoke either an anti-tumor or pro-tumor immune response. P-selectin glycoprotein ligand-1 (PSGL-1) is expressed in macrophages and plays a vital role in synergizing for a more robust anti-tumor response. However, there are few studies about PSGL-1 expression status and clinical value of biological function in cervical cancer.

**Methods:**

In this study, 565 participants were enrolled. PSGL-1 mRNA was detected by real-time quantitative PCR (qPCR) with cervical cytology specimens. The relationship between PSGL-1 and cervical intraepithelial neoplasia in two grades and more (CIN2+) was analyzed, and the optimal cut-off values of PSGL-1 to predict CIN2+ were calculated. In addition, the clinical significance of PSGL-1 in cervical cancer was determined by Kaplan-Meier Cox regression based on the database.

**Results:**

The mean PSGL-1 increased significantly with cervical lesion development, especially compared with CIN2+ (p<0.05). Moreover, the expression of PSGL-1 increased significantly in HPV-16/18 positive and HPV-18 positive, but not in HPV-16 positive and other HR-HPV positive. And then, it demonstrated that the area under the receiver operating characteristic curve (AUC) of PSGL-1 was 0.820, and an optimal cut-off 0.245. Furthermore, the PSGL-1 had the highest odds ratio and highest OR (OR= 8.707; 95% CI (.371-19.321)) for the detection of CIN 2+. In addition, our result also indicated that higher PSGL-1 expression was significantly related to a better prognosis in cervical cancer due to immune cell infiltration.

**Conclusions:**

PSGL-1≥0.245 in cervical cytology specimens is a new auxiliary biomarker of CIN2+, and it may be a promising prognosis predictor and potential immunotherapy target linked with immune infiltration of cervical cancer.

## Introduction

Cervical cancer (CC) is the most common malignancy of the female reproductive system (1). Globally 604,127 emerging cases and 341,831 deaths were reported annually (2). China has 10,9741 new cases and 59,060 deaths annually, an increasing trend (2, 3). Morbidity and mortality are higher in developing countries than in developed countries (4). With the popularization of cervical cancer screening and the advent of the human papillomavirus (HPV) vaccine, the incidence of cervical cancer in developed countries is declining while increasing in developing countries (4). And the age of onset of cervical cancer is younger (1). It has become a significant challenge to female health due to the low coverage of cervical screening and HPV vaccination. Therefore, more effective measures are urgently needed to intervene.

Cervical cancer is mainly squamous cell carcinoma, which is sensitive to radiation. Radiotherapy can be used to achieve the purpose of locally killing tumors. Patients in the middle and advanced stages are treated mainly by combining radiotherapy and chemotherapy. However, the side effects of radiotherapy and chemotherapy are relatively large, and the treatment effect is not well. In recent years, targeted therapy and immunotherapy have been new approaches for treating advanced, recurrent cervical cancer (5). Immunotherapy is an emerging treatment option that might be a novel option to improve the prognosis of these patients. It has recently achieved great preclinical and clinical success through immune checkpoint inhibitors (ICIs) (6–8). Nowadays, ICIs, such as programmed cell death-1(PD-1), programmed cell death-Ligand 1 (PD-L1), and cytotoxic T lymphocyte-associated antigen-4 (CTLA-4), represent the most promising cancer treatment. Anti-PD-1 may have a better curative effect on recurrent or metastatic cervical cancer with its ligand-positive expression. Still, a single drug will likely increase patients’ risk of autoimmune disease. Studies have pointed out that patients are most likely to benefit from pembrolizumab. So it is necessary to identify effective biomarkers other than PD-L1 expression to increase the stability of pembrolizumab efficacy (9, 10). Thus, it is essential to discover a novel target and available predictive biomarker to stratify patients who may benefit from immunotherapy in cervical cancer, such as P-selectin glycoprotein ligand-1 (PSGL-1).

P-selectin glycoprotein ligand-1 (PSGL-1), a principal leukocyte ligand for P-selectin, is also called CD162 and SELPLG. It is mainly expressed on the cell surface of immune and inflammatory cells (11). PSGL-1 is also essential for cell differentiation, as deficiency of PSGL-1 was found to affect the differentiation of myeloid cells and the maturation of lymphocytes (12, 13). PSGL-1 regulates T-cell trafficking into inflamed and lymphoid tissues under steady-state conditions (14, 15). Significantly, PSGL-1 and P-selectin are expressed in macrophages (16). Macrophages secrete many cytokines and chemokines, provoking anti-tumor or pro-tumor immune responses. It is evident that PSGL-1 causes macrophage reprogramming activates T cells, and attracts other immune cells to synergize for a more robust anti-tumor response. Current immunotherapies only provide clinical benefits in about 25% of cancer patients involving T-cell infiltration. By targeting macrophages, which are present in 75% of human tumors, we believe that their additional clinical benefit can be provided to many patients who are under-treated immunotherapy. However, there are few studies about PSGL-1 expression status and biological function in cervical cancer.

This present study aimed to evaluate the predictive value of PSGL-1 expression in cervical cancer. We investigate whether the expression of PSGL-1 can be a valuable biomarker to predict CIN2+ with cervical cytological specimens non-invasively collected. What is more, This study demonstrated the prognosis of PSGL-1. The results provide novel insights into the active role of PSGL-1 in HPV infection and cervical cancer, thereby highlighting a potential mechanistic basis whereby PSGL-1 influences immune cell interaction with tumors.

## Materials and methods

### Patients and study design

This study included 565 participants with cervical specimens from the Fujian Maternity and Child Health Hospital College of Clinical Medicine for Obstetrics & Gynecology and Pediatrics, Fujian Medical University December 2018 to January 2022. The populations must reach the following criteria: 1) age above 20 years; 2) sexually active; 3) no history of cervical cancer, CIN, or HIV infection; and 4) did not undergo a hysterectomy. Or cervix surgery. The Hospital Ethics Committee approved the research of Fujian Provincial Maternity and Children’s Health Hospital, an affiliated hospital of Fujian Medical University (FPMC2021KLRD641), and all individuals participating in this study signed written informed consent.

### Specimen collection and management

Exfoliated cervical cells were collected from cervical canals using a cytobrush. The specimens were collected in vials containing preservation solutions for HPV DNA testing or in bottles of ThinPrep^®^ PreservCyt^®^ solution (Hologic, Waltham, MA, USA) for cytology examination. The specimens for HPV testing need to be stored at −20°C before DNA extraction. And the samples for cytology need to be held at 4°C.

### Liquid-based cytology, HPV genotyping test

Cytological samples were blindly examined, independent of the other assays’ results, by two experienced cytopathologists. The results were reported according to the Bethesda 2001 system (17). If the diagnosis was inconsistent, the cervical specimens were re-evaluated, and a consensus diagnosis was gained. The PCR-RDB HPV genotyping kit (Yaneng Limited Corporation, Shenzhen, China) can discover 18 HR-HPV types and 5 LR-HPV types. All procedures were conducted according to the manufacturer’s instructions provided with the kit (18).

### Histology

According to the guidelines, the colposcopy and needle biopsy in women who are HPV-16/18 positive with or without abnormal cytology [grade higher than atypical squamous cells of undetermined significance (ASC-US)]. Women with a punch biopsy diagnosis with more than high-grade squamous intraepithelial lesions and more (HSIL) got conization by the cold knife or loop electrosurgical excision procedure cone biopsy (LEEP). Specimens were fixed in 10% formalin and routinely processed for paraffin embedding. Then, standard methods cut and stained 4 µm thick tissue sections with hematoxylin and eosin.

### RT-PCR and analysis of PSGL-1

The HPV DNA was extracted from the remaining cervical cells and resuspended in a digestion solution for 3 hours at 56°C. It was incubated at 95°C for ten minutes to inactivate proteinase K. Real-time quantitative PCR (qPCR) was carried out with modifications in the protocol. PSGL-1 PCR was used with primer pairs 5′-ACC CCT GAG TCT ACC ACT GT-3′ and 5′-TCC ATA GCT GCT GAA TCC GT- 3′. β-actin was used as an experimental control for sample quality and adequacy during the PCR process. β-actin PCR was applied with primer pairs 5′-TGA CGT GGA CAT CCG CAA AG-3′ and 5′-CTG GAA GGT GGA CAG CGA GG-3′. Relative levels of PSGL-1 mRNA were quantified by qPCR and calculated by the 2−ΔΔCT method.

### Prognosis analysis

To individualize the prediction of prognosis in cervical cancer patients, we analyzed the OS, DSS, and PFI using the RMS R package (version 6.2-0) and survival package (version 3.2-10) (19). All clinicopathological data were acquired from TCGA-CESC datasets.

### TIMER database analysis

The Tumor Immune Estimation Resource (TIMER2.0) (https://timer.cistrome.org/) is a database used for the analysis of tumor-infiltrating immune cells and various gene expression levels in different types of cancer (20). We assessed PSGL-1 mRNA expression in multiple tumors and the relationship with TILs *via* gene modules. In addition, the relationship between PSGL-1 mRNA expression with gene markers of TILs has been investigated through correlation modules. And the statistical significance of the estimation and correlation of Spearman was analyzed by the correlation module.

### Statistical analysis

The PSGL-1 expression was analyzed in unpaired samples using the Wilcoxon rank-sum test, while paired samples were analyzed using Wilcoxon signed-rank test. Cox regression analysis and Kaplan-Meier analysis were performed to assess the prognostic factors. Using multivariate Cox analysis, we compared the impact of PSGL-1 expression on survival and other clinical characteristics. Furthermore, we conducted ROC analysis using the pROC package to determine whether PSGL-1 expression can accurately distinguish cervical cancer from healthy specimens. Different risks were estimated using odds ratios (ORs) and 95% confidence intervals (CIs).

## Results

### Clinical characteristics and associations between cervical lesions and clinicopathological factors

Analysis of the 565 cervical tissue samples indicated that 136 (24.07%) were standard, 113 (20.00%) were classified as CIN1, 225 (39.83%) were categorized as CIN2/3, and 91 (16.11%) were diagnosed as cancer. The clinicopathologic characteristics of the study population are presented in **Table 1**. Age, cytology, and HPV 16/18 infection, HPV 16 infection, HPV 18 infection, Other HR-HPV infection, HPV A7 infection, and HPV A9 infection were significantly associated with cervical lesions. It demonstrated that abnormal cytology and older age promoted the development of CIN2+. Furthermore, With the development of cervical lesions, PSGL-1 expression increased, especially compared with CIN2+. It suggested that PSGL-1 could predict the development of CIN2+ lesions (p<0.01).

**Table 1:** Clinical characteristics of the participants of the study [N (%)].

Item	Pathology				X^2^	*P*
	Normal (n=136)	CIN 1 (n=113)	CIN 2-3 (n=225)	CC (n=91)		
Age					80.500^a^	<0.001
<30 (N=77)	22 (28.571)	34 (44.156)	19 (24.675)	2 (2.597)		
30-50 (N=382)	94 (24.607)	69 (18.063)	170 (44.503)	49 (12.827)		
≥50 (N=106)	20 (18.868)	10 (9.434)	36 (33.962)	40 (37.736)		
Cervical cytology					215.900^b^	<0.001
NILM (N=167)	98 (58.683)	28 (16.766)	31 (18.563)	10 (5.988)		
ASCUS (N=90)	24 (26.667)	32 (35.556)	32 (35.556)	2 (2.222)		
>ASCUS (N=308)	14 (4.545)	53 (17.208)	162 (52.597)	79 (25.649)		
HPV genetyping						
HPV16/18 infection					71.220	<0.001
HPV16/18 (-)	69 (29.362)	75 (31.915)	81 (34.468)	10 (4.255)		
HPV16/18 (+)	67 (20.303)	38 (11.515)	144 (43.636)	81 (24.545)		
HPV16 infection					95.938	<0.001
HPV16 (-)	105 (32.813)	90 (28.125)	102 (31.875)	23 (7.188)		
HPV16 (+)	31 (12.653)	23 (9.388)	123 (50.204)	68 (27.755)		
HPV18 infection					20.029	<0.001
HPV18 (-)	99 (20.755)	97 (20.335)	203 (42.558)	78 (16.352)		
HPV18 (+)	37 (42.045)	16 (18.182)	22 (25.000)	13 (14.773)		
Other HR-HPV infection					53.011	<0.001
Negative	67 (22.945)	31 (10.616)	123 (42.123)	71 (24.315)		
Positive	69 (25.275)	82 (30.037)	102 (37.363)	20 (7.326)		
HPV A5/6 infection					4.140	0.247
Negative	127 (93.382)	105 (92.920)	218 (96.889)	88 (96.703)		
Positive	9 (6.618)	8 (7.080)	7 (3.111)	3 (3.297)		
HPV A7 infection					26.876	<0.001
Negative	87 (63.971)	92 (81.416)	194 (86.222)	75 (82.418)		
Positive	49 (36.029)	21 (18.584)	31 (13.778)	16 (17.582)		
HPV A9 infection					53.690	<0.001
Negative	60 (44.118)	31 (27.434)	26 (11.556)	15 (16.484)		
Positive	76 (55.882)	82 (72.566)	199 (88.444)	76 (83.516)		
SELPLG	0.216±0.025	0.321±0.047	0.687±0.044	0.809±0.073	626.233	<0.001

CIN, Cervical Intraepithelial Neoplasia; NILM, negative for intraepithelial lesion or malignanc; ASC-US, atypical squamous cells of undetermined significance; CC, cervical cancer. *P* < 0.05.

a-Age group: two-by-two comparison within the group,

a *P*<0.0167; b-Cervical cytology group: two-by-two comparison within the group,

b *P*<0.0167.

**Figure 1:**
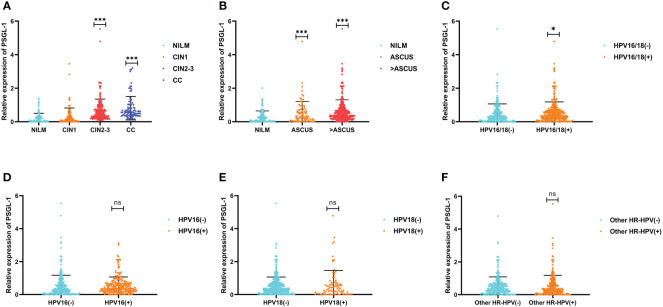
Expression patterns of PSGL-1 mRNA in cervical lesions. **(A)** The PSGL-1 expression increased with the development of cervical lesions. **(B)** PSGL-1 expression increased with the development of cervical cytology. **(C–F)** PSGL-1 expression in different HR-HPV genotyping. Analysis between two groups: Wilcoxon Rank sum test; NS: P= 0.05 or higher; *P < 0.05; ***P < 0.001.

The relation between PSGL-1 with cervical lesions and cytology was summarized in **Figures 1A, B**. We discovered a significant difference in the means of PSGL-1 in four groups of cervical lesions (p<0.001, ANOVA test) (**Figure 1A**). Also, PSGL-1 expression increased significantly in the ASCUS group and higher ASCUS group. Furthermore, we compared the expression of PSGL-1 among different HPV genotyping (**Figures 1C–F**). It revealed that the expression of PSGL-1 increased significantly in HPV-16/18 positive and HPV-18 positive. However, no significant difference was found between HPV-16 positive and other HR-HPV positives.

### PSGL-1 is a valuable predictor for the detection of ≥CIN2

**Figure 2:**
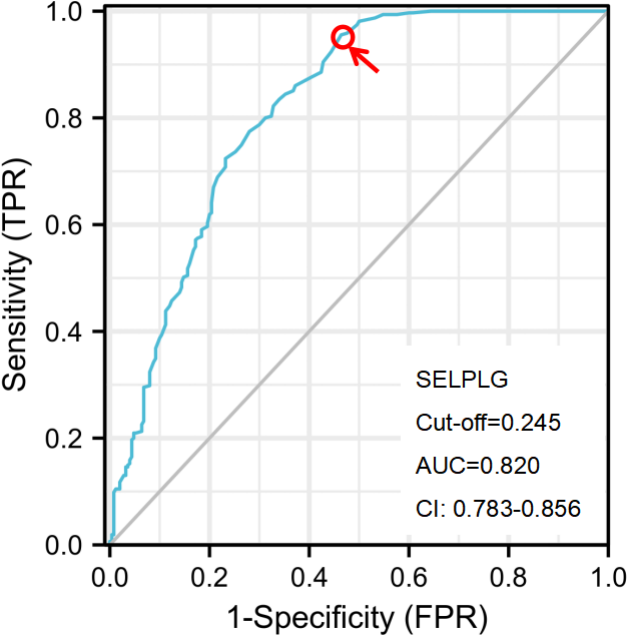
The ROC curve analysis of the PSGL-1 for identifying CIN2+ disease. The optimal PSGL-1 expression used to predict CIN2+ was calculated according to the ROC curve. CIN, cervical intraepithelial neoplasia; NILM, negative for intraepithelial lesion or malignancy; CC, cervical cancer; AUC, the area under the ROC curve; ROC, receiver operator characteristic.

To predict CIN2+ lesions, ROC analyses of PSGL-1 mRNA expression were performed. PSGL-1 mRNA expression was evaluated individually (**Figure 2**). We observed that AUC for PSGL-1 mRNA expression was 0.820. Also, an optimal cut-off of PSGL-1 mRNA expression was 0.245. As shown in **Table 2**, the independent factors associated with the diagnosis of CIN2+ were calculated in the multiple logistic regressions. It is demonstrated that the positivity for PSGL-1 mRNA expression had the highest OR [OR= 8.707; 95% CI (.371-19.321)], followed by HPV genotyping [OR = 4.198; 95% CI (2.001-10.621)], TCT [OR = 3.651; 95% (2.140-8.336)] and age [OR = 2.210; 95% CI (1.130-6.023)].

**Table 2:** Independent predictors for detection of CIN2+ lesions.

	Category	N	OR	95% CI	P
Age	<50	452	1	Reference	0.005
	≥50	113	2.210	1.130-6.023	
TCT	<ASCUS	167	1	Reference	<0.001
	≥ASCUS	398	3.651	2.140-8.336	
HPV genotyping	non-16/18(+)	273	1	Reference	<0.001
	16/18(+)	330	4.198	2.001-10.621	
SELPLG	High (≥0.245)	346	1	Reference	<0.001
	Low	219	8.707	3.371-19.321	

The cutoff of PMR values were defined by ROC curve analysis.

TCT, Thinprep cytologic test; ASC-US, atypical squamous cells of undetermined significance; OR, odds ratio; 95% CI, 95% confidence interval.

### Higher PSGL-1 expression predicts a better prognosis in cervical cancer

Based on TCGA-CESC data sets, a Kaplan-Meier survival analysis was conducted to investigate the role of PSGL-1 mRNA expression in cervical cancer survival. The clinical characteristics of cervical cancers are shown in **Table S1**.

**Figure 3:**
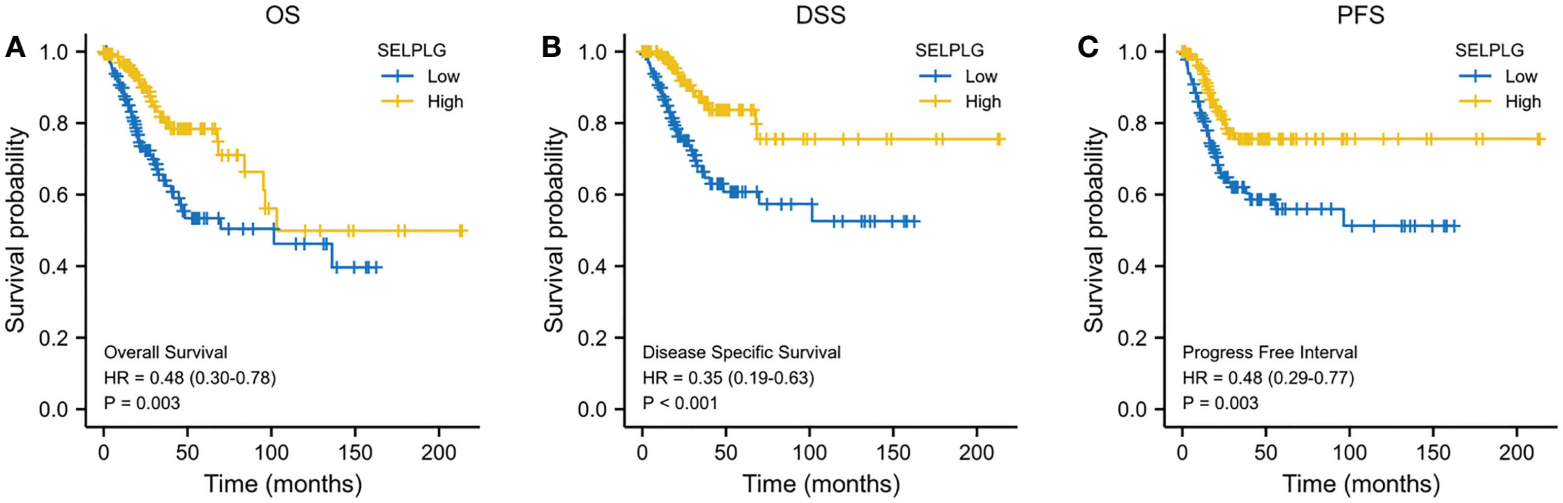
Decreased PSGL-1 mRNA levels predict a poor prognosis in cervical cancer: **(A–C)** Survival curves showed OS, DSS, and PFI rates of CESC patients with high PSGL-1 expression.

We evaluated the impact of PSGL-1 mRNA expression on the prognosis. As shown in **Figure 3**, higher PSGL-1 mRNA expression was significantly related to better overall survival (OS) (HR = 0.48, 95% CI = 0.30-0.78, p = 0.003, **Figure 3A**). Similarly, we found a significant correlation between higher PSGL-1 mRNA expression and better disease-specific survival (DSS)(HR = 0.35, 95% CI = 0.19-0.63, p < 0.001, **Figure 3B**) and progression-free interval (PFI) (HR = 0.48, 95% CI = 0.29-0.77, p = 0.003, **Figure 3C**).

### Association between immune infiltration and PSGL-1 expression in cervical cancer

**Figure 4:**
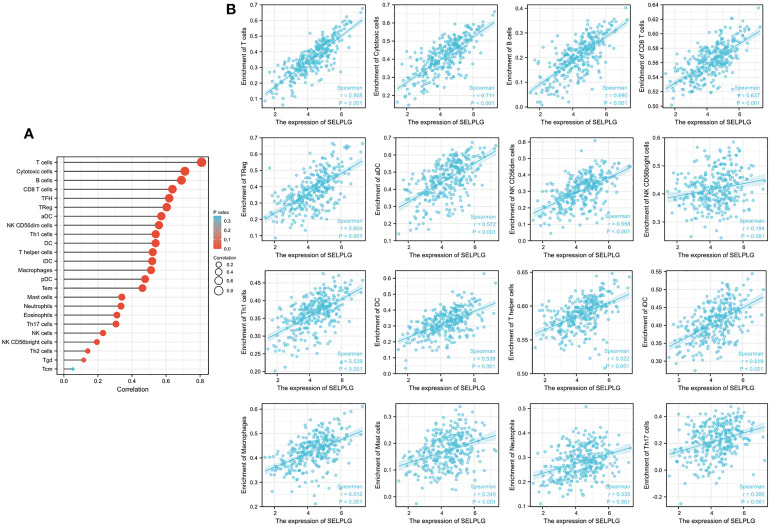
Correlation of PSGL-1 expression with immune infiltration in cervical cancer. **(A)** Correlation between the expression of PSGL-1 and the abundance of TILs in cervical cancer. **(B)** Correlation of PSGL-1 expression with infiltration levels of T cell, cytotoxic cell, B cell, CD8+ T cell, T-cell regulatory, aDC, NK CD56dim cells, NK CD56bright cells, Th1cells, DC, T helper cells, and aDC in cervical cancer available at TIMER2.0 database. TILs, tumor-infiltrating lymphocytes; TIMER2.0, Tumor Immune Estimation Resource. Color images are available online.

Immune infiltration is a crucial factor related to tumor progression. In cervical cancer, PSGL-1 expression was associated with immune cell infiltration levels using TIMER platforms. **Figure 4** shows a strong correlation between PSGL-1 expression level and TIL abundance (**Figure 4A**). For instance, the PSGL-1 expression level was strongly correlated with infiltrating degree of T cell (rho = 0.808), cytotoxic cell (rho = 0.711), B cell (rho = 0.690), CD8+ T cell (rho = 0.637), T-cell regulatory (rho = 0.604), aDC (rho = 0.572), NK CD56dim cells (rho = 0.556), NK CD56bright cells (rho = 0.556), Th1cells (rho = 0.539), DC (rho = 0.538), T helper cells(rho = 0.522), and aDC (rho = 0.519) (**Figure 4B**). All the p-values were below 0.001. These results demonstrated that PSGL-1 expression level plays an essential role in the immune infiltration of cervical cancer.

**Figure 5:**
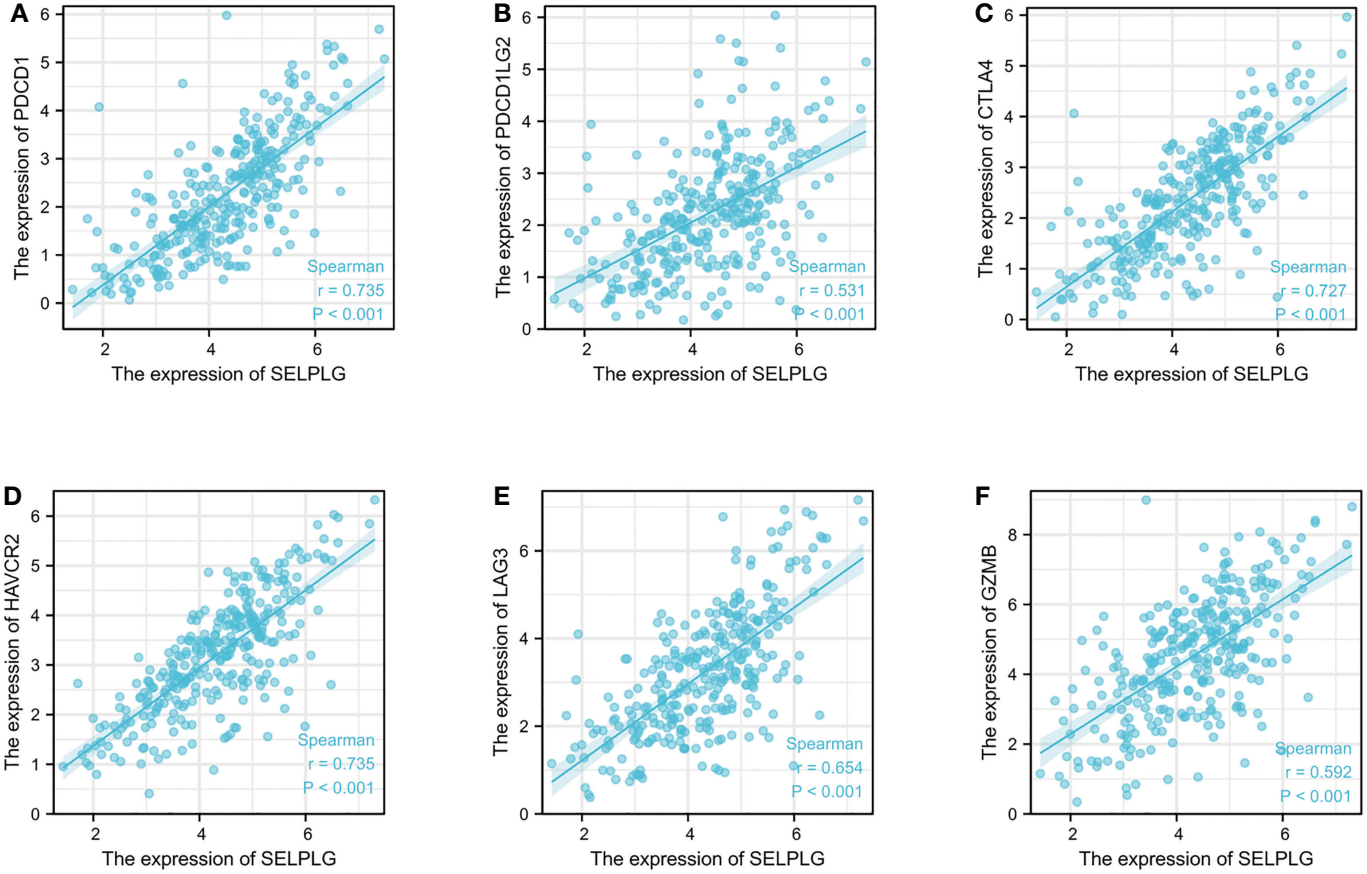
PSGL-1 expression correlated with immunoinhibitors in cervical cancer. **(A–F)**: PDCD1, PDCD1LG2, CTLA4, HAVCR2, LAG3, and GZMB in cervical cancer is available in the TISIDB database.

In addition, this research revealed that PSGL-1 expression level was significantly connected with immunoinhibitors (**Figures 5A–F**). PSGL-1 expression level was significantly with immunoinhibitors, such as PDCD1 (rho = 0.735), PDCD1LG2 (rho = 0.531), CTLA4 (rho = 0.727), HAVCR2 (rho = 0.735), LAG3 (rho = 0.654) and GZMB (rho = 0.592).

## Discussion

This study demonstrated that cervical specimens with over-expression of PSGL-1 showed a significant association with cervical high-grade squamous lesions. Thus, this study may provide information with a novel and valuable biomarker to predict the pathogenesis and development of CIN2+ with no-invasive methods. In addition, the study revealed that high expression of PSGL-1 was significantly associated with HPV-18 infection. Intriguing, it demonstrated that increased expression of PSGL-1 suggested a better prognosis, which is contrary to our perception. We further explored and found a possible mechanism by which PSGL-1 causes this paradoxical phenomenon.

In this study, PSGL-1 expression increased significantly with the development of the cervical lesion. Moreover, PSGL-1 mRNA expression could be a valuable biomarker to predict CIN2+. The AUC for PSGL-1 mRNA expression was 0.820, and an optimal cut-off of PSGL-1 mRNA expression was 0.245. Also, it is demonstrated that the positivity for PSGL-1 mRNA expression had the highest OR (OR= 8.707; 95% CI (.371-19.321)). We suggested cervical specimens from non-invasive procedures and measurement of PSGL-1 mRNA expression as a valuable biomarker to predict CIN2+. Our results may offer a helpful basis for the accurate cytological diagnosis of cervical pre-malignancy and cancer in future transitional research.

Furthermore, we also revealed that the expression of PSGL-1 increased significantly in HPV-16/18 positive and HPV-18 positive. However, no significant difference was found between HPV-16 positive and other HR-HPV positives. It has been reported that the etiology of cervical cancer and precancerous lesions mainly due to the persistent infection of one or several HR-HPV (21, 22). And HPV16/18 accounts for most of the HPV-positive cases (23). This study suggests that PSGL-1 mRNA expression may be an auxiliary indicator for HPV16/18 infection.

PSGL-1 is expressed in almost all leukocytes and binds to and acts on P-selectin in the presence of Ca2+. The binding of PSGL-1 to P-selectin mediates the adhesion of leukocytes to endothelial cells and participates in the adhesion of leukocytes to platelets. Recently, it was reported that PSGL-1 binds other immune inhibitors, including Siglecs and V-domain Ig suppressor of T-cell activation (VISTA) (24, 25). PSGL-1 has been shown to suppress immune responses in various disease models. Julia M DeRogatis et al. (26)pointed out that targeted PD-1 therapy in PSGL-1-deficient tumor-bearing mice can enhance anti-tumor immunity and slow the growth of melanoma tumors. Tumors and white blood cells have many similarities in migration and spread (27). Therefore, tumor cell-derived PSGL-1 may also play an essential role in the biological behavior of tumor cells, such as infiltration, migration, etc. For instance, Dimitroff et al. (28). found that PSGL-1 is involved in the process of prostate cancer bone metastasis. Prostate cancer cells with high expression of PSGL-1 can metastasize far away. Their ability to metastasize to bone is significantly higher than other tissues such as the lung, lymph node, and liver. Similarly, Heidemann et al. (29). based on animal experiments, that PSGL-1 contributes to the distant metastasis of small cell lung cancer. Hoos et al. (30) have shown that PSGL-1 derived from tumor cells can promote metastasis by binding to selectins on the surface of vascular endothelium, platelets, and leukocytes. Furthermore, metastatic tumor cells promote the expression of selectin from monocyte-macrophage through the recruitment of monocytes so that tumor cells can survive, extravasate, and metastasize more efficiently. In addition, P-selectin and its ligand PSGL-1 play essential roles in hematopoiesis, T-cell activation, tumor growth, and metastasis (31, 32).

Our study revealed that higher PSGL-1 mRNA expression was significantly related to better overall survival (OS) (HR = 0.48, 95% CI = 0.30-0.78, p = 0.003). Similarly, we found a significant correlation between higher PSGL-1 mRNA expression and better disease-specific survival (DSS)(HR = 0.35, 95% CI = 0.19-0.63, p < 0.001) and progression-free interval (PFI) (HR = 0.48, 95% CI = 0.29-0.77, p = 0.003). Interestingly, we found that the expression of PSGL-1 was significantly associated with prognosis, which shows that higher PSGL-1 expression predicts a better prognosis, contrary to our general knowledge. This may be closely related to the role of PSGL-1. It is reported that PSGL-1 is a mucin-like sialylated surface membrane-associated glycoprotein. As a ligand for P-selectin, E-selectin, and L-selectin, it mainly mediates cell adhesion, lymphocyte homing, and regulation of leukocyte rolling (12, 13).

We analyzed the correlation of PSGL-1 with other immune cell infiltration using the TIMER database. We found that PSGL-1 expression level was strongly correlated with infiltrating degree of T cell, CD8+ T cell, cytotoxic cell, B cell, T-cell regulatory, aDC, NK CD56dim cells, NK CD56bright cells, Th1cells, DC, T helper cells, and aDC. These results suggest that PSGL-1 is essential in regulating the tumor immune microenvironment. It is well known now that CD8 T cells play a central role in mediating anti-tumor immunity. Their effector CTLs eliminate tumor cells by recognizing tumor-associated antigens presented on primary histocompatibility complex class I (MHCI) by their expressed T cell receptor (TCR). Studies have reported that infiltration of T cells, especially CD8 T cells, into the tumor microenvironment (TME) demonstrated a good prognosis in breast, lung, melanoma, colorectal, and brain cancer (33, 34). Insufficient T cell priming likely contributes to cold tumors (no T cell infiltration in TME) and unresponsiveness to immune checkpoint blockade (ICB) therapy (35). Apart from this, we found that PSGL-1 expression level was significantly connected with immunoinhibitors, such as PDCD1, PDCD1LG2, CTLA4, HAVCR2, LAG3, and GZMB. This evidence suggests that PSGL-1 may enhance the anti-tumor effect by increasing the infiltration of anti-tumor immune cells and promoting the expression of the immune checkpoint. Therefore, this study further pointed out that PSGL-1 can be used as a new target for cervical cancer treatment. Also, it provides a new research direction for the combined treatment of cervical cancer with immunotherapy.

PSGL-1 and P-selectin are expressed in macrophages. It is evident that PSGL-1 causes macrophage reprogramming activates T cells, and attracts other immune cells to synergize for a more robust anti-tumor response. Therefore, PSGL-1 is expected to become an effective immune checkpoint inhibitor. Their additional clinical benefit can be provided to many patients who are under-treated immunotherapy. However, these are a few studies to test and verify the expression of PSGL-1 in cervical cancer. Our study demonstrated the correlation of PSGL-1 protein expression with high-grade cervical lesions and hypothesized that PSGL-1 expression might be associated with the pathogenesis of high-grade cervical lesions and cervical cancer. This study fills the gaps in the primary and clinical research of PSGL-1 protein in the field of cervical cancer, provides theoretical support for the prevention, diagnosis, and treatment of cervical cancer, and also seeks new targets for the treatment of cervical cancer. It provides valuable clinical data for PSGL-1 in human biological function research, with good innovation and progress. However, there are some limitations, as follows. Firstly, the specimen size needed to be more significant. Increasing the sample size may be necessary for future studies. Secondly, in this study, we did not use our data to verify the relationship between PSGL-1 and the prognosis of cervical cancer, which is worth further studying. It can better help us make disease diagnoses and prognosis judgments, providing a more accurate clinical basis for clinically precise and individualized treatment.

In conclusion, these results revealed that PSGL-1 expression significantly increased in cervical cytology is strongly related to a higher grade of cervical lesions. PSGL-1 mRNA expression can be a suitable biomarker to predict CIN2+. Moreover, higher PSGL-1 expression predicts a better prognosis in cervical cancer due to increased infiltration of anti-tumor immune cells and promotion of the expression of the immune checkpoint. These results may provide a valuable research direction for the combined treatment of cervical cancer with immunotherapy.

## Data availability statement

The datasets presented in this study can be found in online repositories. The names of the repository/repositories and accession number(s) can be found in the article/**Supplementary Material**.

## Ethics statement

The studies involving human participants were reviewed and approved by Fujian Provincial Maternity and Children’s Health Hospital, an affiliated hospital of Fujian Medical University(FPMC2021KLRD641). The patients/participants provided their written informed consent to participate in this study.

## Author contributions

YL was responsible for the data acquisition, analysis, and manuscript drafting. SH and YQ participated in data acquisition, research, and interpretation. LX and JJ participated in drafting the manuscript and troubleshooting. HL and ZC participated in its design and coordination. All authors contributed to the article and approved the submitted version.
