# Supplementary material for: PSGL-1 is a novel tumor microenvironment prognostic biomarker with cervical high-grade squamous lesions and more
Source: Front Oncol. 2023 Mar 8;13:1052201. doi: 10.3389/fonc.2023.1052201 (PMC10030516; doi:10.3389/fonc.2023.1052201)
Supplement: Supplementary file 1 [file Table_1.pdf]

Table S1. Clinical characteristics of the cervical cancers in TCGA datasets

Characteristic	Low expression of SELPLG	High expression of SELPLG	p
n	153	153	
T stage, n (%)			0.276
T1	66 (27.2%)	74 (30.5%)	
T2	29 (11.9%)	43 (17.7%)	
T3	13 (5.3%)	8 (3.3%)	
T4	6 (2.5%)	4 (1.6%)	
N stage, n (%)			0.552
N0	56 (28.7%)	78 (40%)	
N1	29 (14.9%)	32 (16.4%)	
M stage, n (%)			0.530
M0	49 (38.6%)	67 (52.8%)	
M1	6 (4.7%)	5 (3.9%)	
Age, median (IQR)	47 (39, 57)	46 (38, 56)	0.448
